# Activation of Adenosine A3 Receptor Alleviates TNF-****α****-Induced Inflammation through Inhibition of the NF-**κ**B Signaling Pathway in Human Colonic Epithelial Cells

**DOI:** 10.1155/2014/818251

**Published:** 2014-04-22

**Authors:** Tianhua Ren, Yumei Qiu, Weiyun Wu, Xiao Feng, Shicai Ye, Zhuang Wang, Ting Tian, Yanting He, Caiyuan Yu, Yu Zhou

**Affiliations:** Department of Gastroenterology, The Affiliated Hospital of Guangdong Medical College, Zhanjiang 524001, China

## Abstract

To investigate the expression of adenosine A3 receptor (A3AR) in human colonic epithelial cells and the effects of A3AR activation on tumor necrosis factor alpha (TNF-**α**-) induced inflammation in order to determine its mechanism of action in human colonic epithelial cells, human colonic epithelial cells (HT-29 cells) were treated with different concentrations of 2-Cl-IB-MECA prior to TNF-**α** stimulation, followed by analysis of NF-**κ**B signaling pathway activation and downstream IL-8 and IL-1**β** production. A3AR mRNA and protein were expressed in HT-29 cells and not altered by changes in TNF-**α** or 2-Cl-IB-MECA. Pretreatment with 2-Cl-IB-MECA prior to stimulation with TNF-**α** attenuated NF-**κ**B p65 nuclear translocation as p65 protein decreased in the nucleus of cells and increased in the cytoplasm, inhibited the degradation of I**κ**B-**α**, and reduced phosphorylated-I**κ**B-**α** level significantly, compared to TNF-**α**-only-treated groups. Furthermore, 2-Cl-IB-MECA significantly decreased TNF-**α**-stimulated IL-8 and IL-1**β** mRNA expression and secretion, compared to the TNF-**α**-only treated group. These results confirm that A3AR is expressed in human colonic epithelial cells and demonstrate that its activation has an anti-inflammatory effect, through the inhibition of NF-**κ**B signaling pathway, which leads to inhibition of downstream IL-8 and IL-1**β** expression. Therefore, A3AR activation may be a potential treatment for gut inflammatory diseases such as inflammatory bowel disease.

## 1. Introduction

Adenosine is released from a variety of cells in response to inflammatory and metabolic stress and exerts its physiological functions by interacting with 4 subtypes of adenosine receptors, including A1, A2A, A2B, and A3 receptor [1, 2]. Adenosine A3 receptor (A3AR) is a G_*i*_-protein-coupled receptor that, when being activated, decreases intracellular cAMP levels [3]. The* A3AR* gene contains 2 exons that are separated by a single intron of approximately 2.2 kb, located on human chromosome 1p21-p13 [4]. There is extensive evidence for the involvement of A3AR in a number of pathophysiological processes including ischemic conditions of the central nervous system and heart and inflammatory states, such as rheumatoid arthritis (RA), Crohn's disease, and tumors [4, 5]. Therefore, A3AR is an important target for a number of inflammatory, neoplastic, and neurodegenerative conditions.

Several studies have provided evidence to support the theory that activation of A3AR is crucial for anti-inflammatory responses. Ochaion et al. found that, in RA patients, the A3AR agonist CF502 mediated an anti-inflammatory effect by inhibiting the PI3K, PKB/Akt, and nuclear factor-kappaB (NF-*κ*B) signaling pathway [6]. Lee et al. demonstrated that, in murine BV2 microglial cells, activation of A3AR suppresses lipopolysaccharide- (LPS-) induced tumor necrosis factor-*α* (TNF-*α*) production through inhibition of PI3-kinase/Akt and NF-*κ*B activation. The authors of the study proposed that selective ligands of A3AR may have therapeutic potential for the modulation and possible treatment of brain inflammation [7]. In sepsis, A3AR knockout mice had significantly higher levels of plasma TNF-*α*, increased mRNA encoding proinflammatory cytokines, and enhanced nuclear translocation of NF-*κ*B in their renal cortices compared with A3AR wild type (A3AR WT) mice. A3AR WT mice treated with the A3AR agonist IB-MECA showed acutely improved renal and hepatic function, indicating that A3AR activation confers significant protection from murine septic peritonitis primarily by attenuating the hyperacute inflammatory response in sepsis [8].

However, the role and mechanism of A3AR in the human colonic epithelial inflammatory response are not clear. Colonic epithelial cells, which act as sentinels of the mucosal immune system, are critical to the barrier and absorptive functions of the colon [9, 10]. Human colonic epithelial cells express numerous inflammatory molecules, including cytokines, chemokines, and receptors, which allow them to communicate with the immune system [11]. NF-*κ*B is critical for the maintenance of epithelial barrier function and modulation of immune responses. The activation of the RelA subunit (p65) is a major point in the classical NF-*κ*B signaling pathway, which is required for transactivation of gene expression [12, 13]. NF-*κ*B is sequestered in the cytoplasm of resting cells by inhibitory proteins belonging to the NF-*κ*B inhibitor (I*κ*B) family. Following cell exposure to various stimuli, including LPS and TNF-*α*, I*κ*B-*α* is first phosphorylated and rapidly degraded in the proteasomes, allowing NF-*κ*B nuclear translocation and gene activation [14]. Increased activation of NF-*κ*B triggers proinflammatory cytokine expression, resulting in exacerbated colonic inflammatory responses; therefore, suppression of NF-*κ*B signaling may inhibit disease activity in murine models of colitis [15]. It has been reported that adenosine is a negative regulator of NF-*κ*B signaling, resulting in a reduction in interleukin (IL)-8 expression and secretion in human intestinal epithelial cells [16].

In this study, we investigated the effect of A3AR activation on TNF-*α*-induced inflammatory responses in human colonic epithelial cells. Our results showed that activation of A3AR alleviated TNF-*α*-induced inflammation through inhibition of NF-*κ*B signaling pathway and downstream proinflammatory cytokines IL-8 and IL-1*β* production in HT-29 cells, suggesting that A3AR may play an important role in the regulation of colonic epithelial inflammation.

## 2. Materials and Methods

### 2.1. Reagents

RPMI1640 medium and fetal bovine serum (FBS) were purchased from GIBCO (MD, USA); human tumor necrosis factor-alpha (hTNF-*α*) was purchased from Cell Signaling Technology (MA, USA); antibodies to A3AR, NF-*κ*B p65, I*κ*B-*α*, and phosphorylated-I*κ*B-*α* were purchased from Santa Cruz Biotechnology (CA, USA); secondary antibodies horseradish peroxidase-conjugated goat IgG were purchased from Beyotime (Jiangsu, China). 1-[2-Chloro-6-[[(3-iodophenyl)methyl]amino]-9*H*-purin-9-yl]-1-deoxy-*N*-methyl-*β*-D-ribofuranuronamide (2-Cl-IB-MECA) was purchased from Tocris Bioscience (Bristol, UK), and a stock solution of 100 mM was prepared in DMSO. The Cy 3-conjugated AffiniPure donkey anti-mouse IgG and fluorescein isothiocyanate- (FITC-) conjugated AffiniPure donkey anti-rabbit IgG were purchased from Jackson ImmunoResearch Laboratories (PA, USA); RNAiso Plus, PrimeScript RT MasterMix Perfect Real Time, and SYBR Premix Ex Taq II (Perfect Real Time) were purchased from Takara (Dalian, China). Enzyme-linked immunosorbent assay (ELISA) kits were purchased from Sangon Biotech (Shanghai, China).

### 2.2. Cell Culture and Treatments

HT-29 cells were cultured in RPMI 1640 medium supplemented with 10% FBS, 100 IU/mL penicillin, and 100 *μ*g/mL streptomycin at 37°C and 5% CO_2 _in a humidified atmosphere. In all cases, cells were cultured in serum and antibiotic-free medium for 12 h prior to experimentation. For some experiments, cells were pretreated with various concentrations of 2-Cl-IB-MECA for 30 min and then stimulated with TNF-*α* (10 ng/mL) for the indicated times. Prior to use, the 2-Cl-IB-MECA was dissolved in DMSO at appropriate concentrations, and the final concentration of DMSO in each sample did not exceed 0.1%. The negative control (NC) group received no treatment.

### 2.3. CCK-8 Viability Assay

Viability was estimated using the CCK-8 assay. HT-29 cells were seeded into a 96-well plate, cultured with various concentrations (5–1000 nM) of 2-Cl-IB-MECA for 12 h, and finally incubated with CCK-8 reagent for 1 h at 37°C. The absorbance was detected at 450 nm on a microplate reader.

### 2.4. Immunofluorescence (IF) Analysis

IF staining was performed according to standard protocols. Briefly, cells were cultured for 24 h on a slide in a 24-well plate, followed by pretreatment with 30 nM 2-Cl-IB-MECA for 30 min before TNF-*α* (10 ng/mL) stimulation for the indicated times at 37°C. After fixation for 15 min with 4% paraformaldehyde, cells were rendered permeable by incubation in 0.1% Triton X-100 for 5 min, then blocked with 10% donkey serum albumin at room temperature (RT) for 30 min, and incubated with primary antibodies against A3AR (rabbit anti-human, 1 : 100), NF-*κ*B p65 (mouse anti-human, 1 : 100), I*κ*B-*α* (mouse anti-human, 1 : 100), or phosphorylated-I*κ*B-*α* (mouse anti-human, 1 : 100) overnight at 4°C. After rinsing three times in PBS, the cells were incubated with secondary antibodies (donkey anti-rabbit FITC or donkey anti-mouse Cy 3, 1 : 200) for 1 h at RT in the dark and then washed 3 times with PBS. Finally, coverslips were mounted on slides using fluorescent mounting medium with 4′6-diamidino-2-phenylindole (DAPI) to counterstain the nuclei. The staining was evaluated on a Leica converted fluorescence microscope.

### 2.5. Real-Time Quantitative Reverse Transcription Polymerase Chain Reaction (qRT-PCR)

For qRT-PCR experiments, total RNA from HT-29 cells was extracted using RNAiso Plus following the manufacturer's instructions. Five hundred nanograms of total RNA was converted to cDNA and qRT-PCR reactions were performed on a LightCycler system (Roche Diagnostics, USA) in a 96-well format over 45 cycles with denaturation at 95°C for 10 s and annealing at 58°C for 20 s. The primers were synthesized by Sangon Biotech (Shanghai, China) and are listed in Table 1.

### 2.6. Western Blot Analysis

Cytoplasmic or nuclear extracts from HT-29 cells with various treatments were collected separately using a nuclear and cytoplasmic protein extraction kit (Sangon; China) according to the manufacturer's protocol. The total protein from HT-29 cells was collected using cell lysis buffer containing phenylmethylsulfonyl fluoride (Beyotime, China). The protein concentrations were measured by using a BCA protein assay kit (Beyotime, China). Equal amounts of protein were separated by SDS-polyacrylamide gel electrophoresis (SDS-PAGE) and then transferred onto a polyvinylidene fluoride membrane (PVDF, Millipore, USA). The membrane was blocked for 1 h with 5% fat-free milk at RT and then incubated with primary antibodies against A3AR (rabbit anti-human, 1 : 800), NF-*κ*B p65 (mouse anti-human, 1 : 600), I*κ*B-*α* (mouse anti-human, 1 : 400), or phosphorylated-I*κ*B-*α* (mouse anti-human, 1 : 400), tubulin (mouse anti-human, 1 : 1000), and *β*-actin (mouse anti-human, 1 : 1000) at 4°C overnight. Membranes were washed three times with Tris-buffered saline with Tween-20 (TBS-T) and incubated with corresponding secondary antibodies (horseradish peroxidase- (HRP-) labeled goat anti-mouse IgG, HRP-labeled goat anti-rabbit IgG, 1 : 1000; Beyotime, China) for 1 h at RT. Signals were detected with ECL detection reagent (Beyotime, China). The images were obtained on Kodak film and quantified by using Quantity One software (Bio-Rad, USA).

### 2.7. ELISA

IL-8 and IL-1*β* protein secretion from HT-29 cells was measured via ELISA. To do this, the cell supernatants were collected after various treatments and cleared by centrifugation in order to remove cell debris. Concentration of IL-8 and IL-1*β* was determined by using ELISA kits according to the manufacturer instructions (Sangon, China) using an ELISA plate reader. For each experiment, three individual wells of each drug concentration were prepared. The concentrations of IL-8 and IL-1*β* released into the medium are expressed as picograms per milliliter (pg/mL).

### 2.8. Statistical Analysis

Data are expressed as means ± standard deviation (SD). Statistical analyses were performed by one-way analysis of variance and considered significant if* P *values were <0.05. Each experiment was performed three times as stated.

## 3. Results

### 3.1. Cell Viability

**Figure 1:**
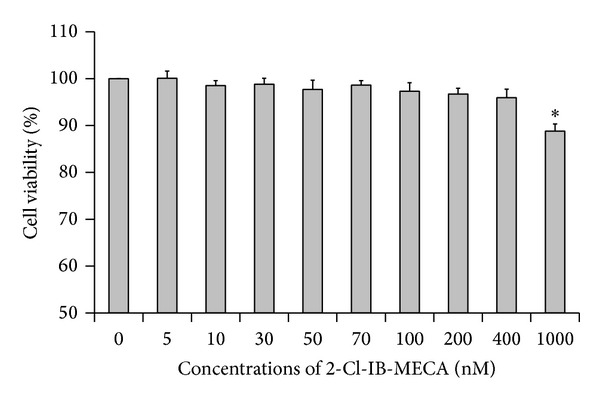
Cell survival rate at various concentrations of 2-Cl-IB-MECA. HT-29 cells were treated with various concentrations of 2-Cl-IB-MECA (5 nM to 1000 nM) for 12 hours, before cell viability was measured by using a CCK-8 assay. Compared with the NC group, the cell viability of each group showed no significant difference (*P* > 0.05), except for the 1000 nM group (*P < 0.05). Data are shown as the mean ± SD from three independent experiments.

To determine whether the A3AR agonist 2-Cl-IB-MECA affected the viability of HT-29 cells, we performed a CCK-8 assay. As shown in Figure 1, no significant effect on cell viability was observed at test concentrations of up to 400 nM and the cell survival rate of each group exceeded 90%. However, when concentration was increased to 1000 nM, the cell viability was significantly downregulated compared to the control group (*P* < 0.05). These data demonstrated that concentrations of 2-Cl-IB-MECA that were less than 400 nM did not affect the rate of HT-29 cell survival. Based on previous reports and our preliminary experiments, the concentration of 2-Cl-IB-MECA used in subsequent studies was 10 nM, 30 nM, and 50 nM.

### 3.2. Location and Qualitative Expression of A3AR, NF-*κ*B p65, I*κ*B-*α*, and Phosphorylated-I*κ*B-*α* in HT-29 Cells Treated with TNF-*α* and/or 2-Cl-IB-MECA

**Figure 2:**
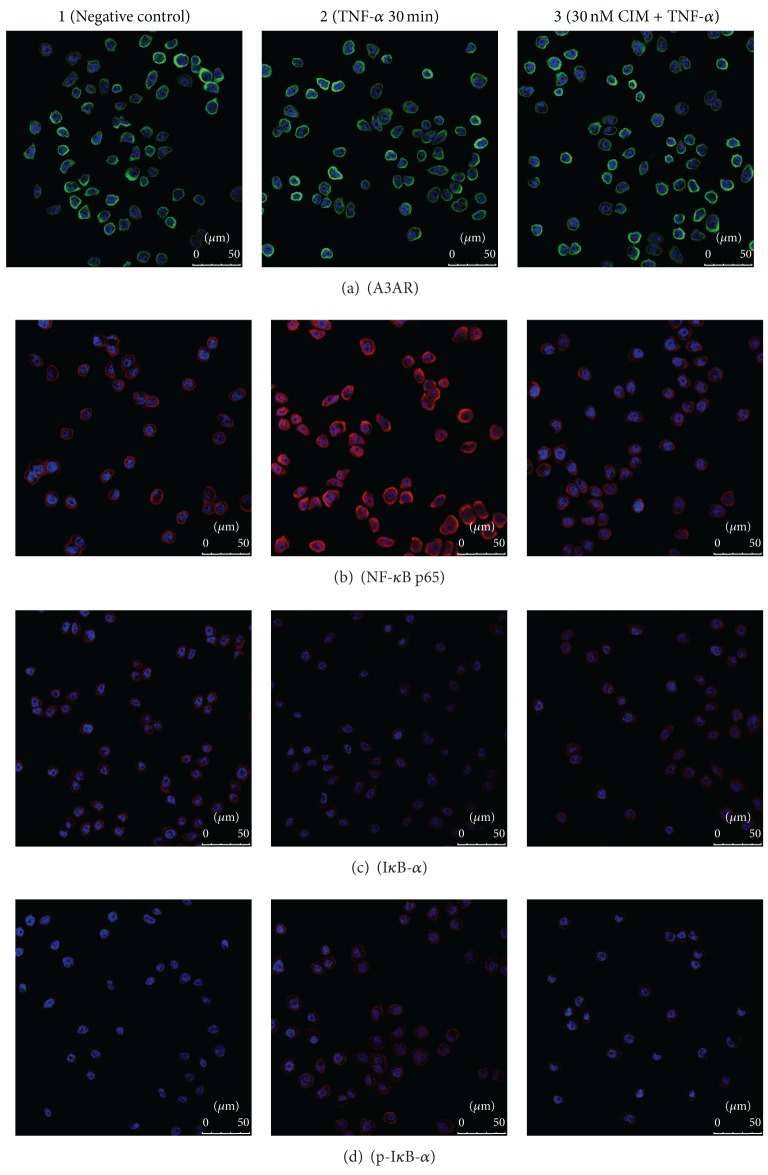
Qualitative expression of A3AR, NF-*κ*B p65, I*κ*B-*α*, and phosphorylated-I*κ*B-*α* (p-I*κ*B-*α*) in HT-29 cells following different treatments. HT-29 cells were treated with 30 nM 2-Cl-IB-MECA (CIM) for 30 min prior to TNF-*α* (10 ng/mL) stimulation for 30 min for the immunofluorescence (IF) assay. IF was performed by using specific antibodies for target proteins and DAPI for counterstaining of nuclei. ((a)1–3) The strong green fluorescence signal representing A3AR was observed mainly on the HT-29 cell membrane. However, cells stimulated by TNF-*α* alone or pretreated with 2-Cl-IB-MECA had no obvious change in A3AR expression. ((b)1–3) In untreated cells, NF-*κ*B p65 was limited to the cytoplasm, and nuclear localization was observed in TNF-*α*-treated HT-29 cells. However, a significant reduction in p65 nuclear translocalization was seen in 2-Cl-IB-MECA + TNF-*α* cells. ((c)1–3) There was a tendency for I*κ*B-*α* to decrease in the cytoplasm of TNF-*α*-treated cells. This effect was inhibited in 2-Cl-IB-MECA + TNF-*α* cells. ((d)1–3) There was a tendency for p-I*κ*B-*α* to increase in the cytoplasm of TNF-*α*-treated cells. This effect was inhibited in 2-Cl-IB-MECA + TNF-*α* cells. Magnification ×600; scale bars indicated 50 *μ*m.

To verify the presence of A3AR, NF-*κ*B p65, I*κ*B-*α*, and phosphorylated-I*κ*B-*α* in HT-29 cells, IF was performed. Representative micrographs from these experiments are shown in Figure 2. Firstly, a strong green fluorescence signal representing A3AR was observed mainly on HT-29 cell membranes, consistent with previous reports that A3AR is a transmembrane receptor. However, TNF-*α*-stimulated cells pretreated with 2-Cl-IB-MECA showed no obvious change in A3AR expression (Figure 2(a)). Secondly, confocal microscopy of NF-*κ*B p65 in HT-29 cells is shown in Figure 2(b). In untreated cells, NF-*κ*B p65 was mainly located in the cytoplasm and was almost absent from the nucleus (Figure 2(b)1). TNF-*α* stimulation induced NF-*κ*B p65 translocation to the nuclei. Translocation of NF-*κ*B p65 towards the nucleus can be observed in Figure 2(b)2. Meanwhile, impaired I*κ*B-*α* expression and the increased expression of phosphorylated-I*κ*B-*α* in the cytoplasm, induced by TNF-*α*, could be observed in Figures 2(c)2 and 2(d)2. Pretreatment with 2-Cl-IB-MECA and subsequent stimulation with TNF-*α* attenuated NF-*κ*B p65 nuclear translocation and suppressed the phosphorylation of I*κ*B-*α* (Figures 2(b)3, 2(c)3, and 2(d)3).

### 3.3. mRNA and Protein Expression of A3AR in HT-29 Cells Treated with TNF-*α* and/or 2-Cl-IB-MECA

**Figure 3:**
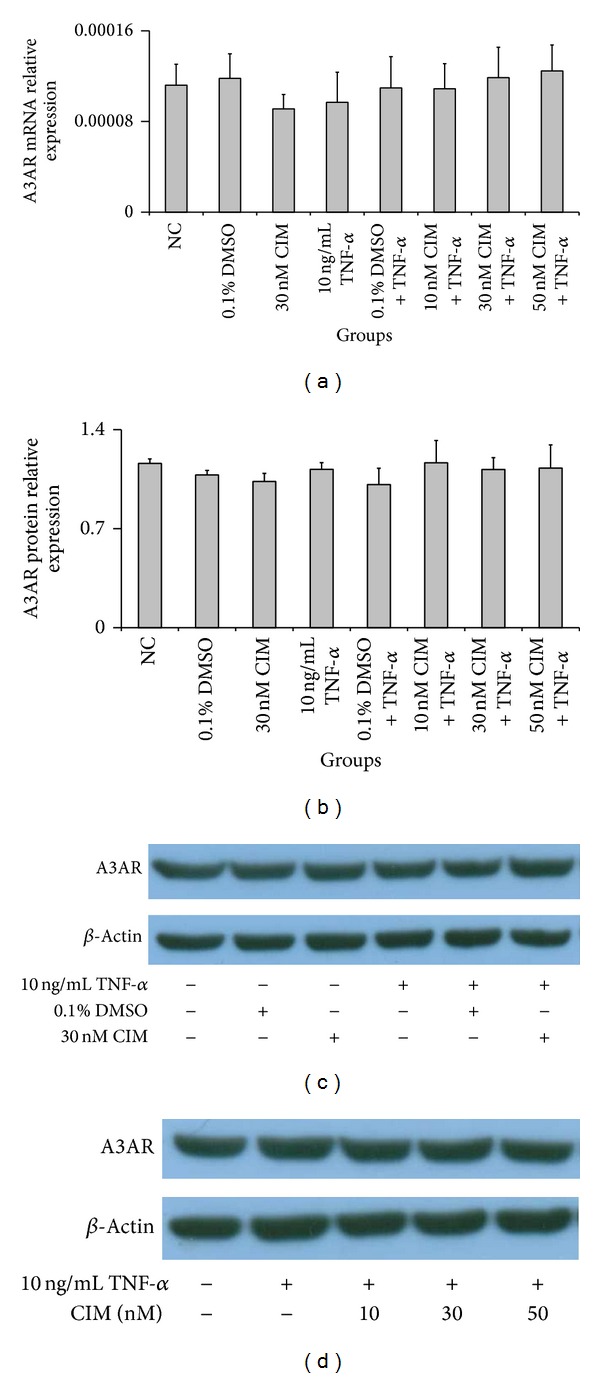
Expression of A3AR mRNA and protein in HT-29 cells. HT-29 cells were treated with various concentrations (10 nM, 30 nM, and 50 nM) of 2-Cl-IB-MECA for 30 min and then incubated with TNF-*α* (10 ng/mL) for 6 h in order to perform mRNA expression assays by qRT-PCR or 18 h for protein expression measurements by western blot. (a, b) Compared with the NC group, the mRNA and protein expression levels of A3AR showed no significant alterations in other groups (*P* > 0.05). (c, d) Representative pictures of A3AR protein expression in indicated groups detected by western blot. Data are shown as the mean ± SD from three independent experiments.

The effects of TNF-*α* and 2-Cl-IB-MECA on the expression of A3AR are shown in Figure 3. The mRNA and protein expression of A3AR showed no significant change among the NC group, TNF-*α*-only-treated group, and 2-Cl-IB-MECA + TNF-*α* groups (*P* > 0.05). These data suggest that neither TNF-*α* nor 2-Cl-IB-MECA can change the expression of A3AR in HT-29 cells, consistent with our IF results.

### 3.4. Effects of 2-Cl-IB-MECA on TNF-*α*-Induced NF-*κ*B Activation

**Figure 4:**
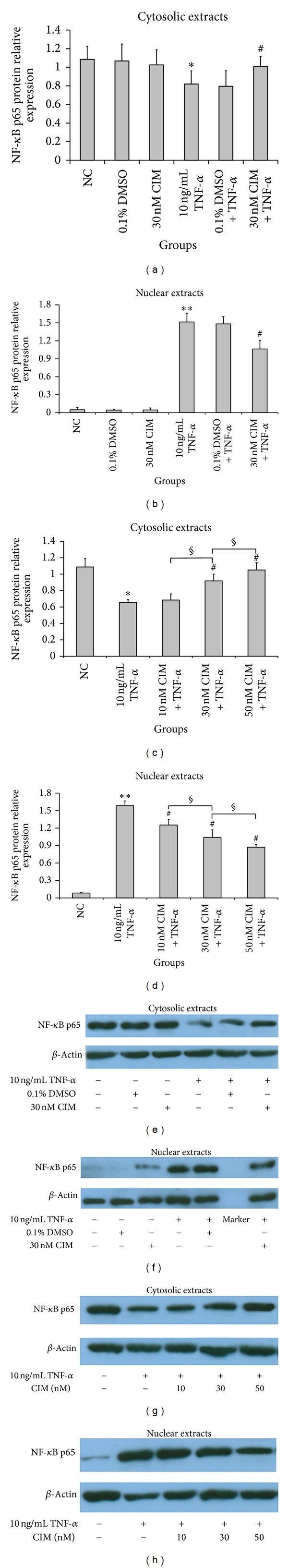
Effect of 2-Cl-IB-MECA on TNF-*α*-induced NF-*κ*B p65 activation in HT-29 cells. HT29 cells were treated with various concentrations (10 nM, 30 nM, and 50 nM) of 2-Cl-IB-MECA for 30 min and then incubated with TNF-*α* (10 ng/mL) for 30 min. After that, cytosolic and nuclear proteins were extracted and analyzed by western blot. (a, b) TNF-*α* resulted in a reduction of NF-*κ*B p65 in cytosolic extracts and an increase of NF-*κ*B p65 in nuclear extracts from HT-29 cells, demonstrating TNF-*α*-induced NF-*κ*B p65 translocation from the cytoplasm to the nucleus. (c, d) Compared with the TNF-*α*-only-treated group, 2-Cl-IB-MECA pretreatment reversed the effects of TNF-*α*, leading to a significant increase in NF-*κ*B p65 in the cytoplasm and a decrease in NF-*κ*B p65 in the nucleus, both in a concentration-dependent manner. Data are shown as the mean ± SD from three independent experiments. (e–h) Representative pictures of NF-*κ*B p65 protein expression in indicated groups detected by western blot. *P < 0.05 and **P < 0.01 compared with the NC group; ^#^ *P* < 0.05 compared with the TNF-*α*-only-treated group; ^§^ *P* < 0.05 between indicated groups.

**Figure 5:**
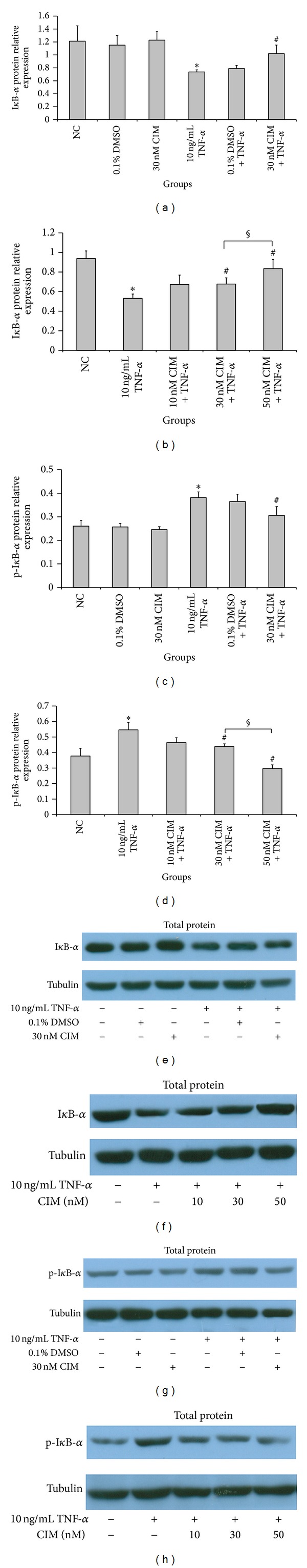
Effect of 2-Cl-IB-MECA on TNF-*α*-induced I*κ*B-*α* and p-I*κ*B-*α* protein expression in HT-29 cells. HT29 cells were treated with various concentrations (10 nM, 30 nM, and 50 nM) of 2-Cl-IB-MECA for 30 min before TNF-*α* (10 ng/mL) stimulation for 30 min. Then total protein was extracted and analyzed by western blot. (a, c) TNF-*α* resulted in a reduction in I*κ*B-*α* expression and an increase in p-I*κ*B-*α* expression in HT-29 cells. (b, d) Compared with the TNF-*α*-only-treated group, 2-Cl-IB-MECA pretreatment enhanced the expression of I*κ*B-*α* and attenuated the expression of p-I*κ*B-*α* in a concentration-dependent manner. (e, f) Representative pictures of I*κ*B-*α* protein expression in indicated groups detected by western blot. (g, h) Representative pictures of p-I*κ*B-*α* protein expression in indicated groups detected by western blot. Data are shown as the mean ± SD from three independent experiments. **P < 0.05 compared with the NC group; ^#^ *P* < 0.05 compared with the TNF-*α*-only-treated group; ^§^ *P* < 0.05 between the indicated groups.

To determine whether A3AR activation participates in TNF-*α*-induced NF-*κ*B activation, we estimated the level of NF-*κ*B p65, I*κ*B-*α*, and phosphorylated-I*κ*B-*α* using western blot after HT29 cells were treated with various concentrations (10 nM, 30 nM, and 50 nM) of 2-Cl-IB-MECA for 30 min and then incubated with TNF-*α* (10 ng/mL) for 30 min. As the results show, NF-*κ*B p65 mainly resided in the cytoplasm and was almost absent from the nucleus (Figure 4.). Stimulation of HT-29 cells with TNF-*α* resulted in a significant shift of NF-*κ*B p65 towards the nucleus, as p65 protein expression increased in the nucleus of the cells and decreased in the cytoplasm. We also observed a decrease in I*κ*B-*α* and an increase in phosphorylated-I*κ*B-*α* expression, compared to the control groups (*P* < 0.05, Figures 4 and 5). Pretreatment with 2-Cl-IB-MECA prior to stimulation with TNF-*α* attenuated NF-*κ*B p65 nuclear translocation, as p65 protein decreased in the nucleus of cells and increased in the cytoplasm, inhibited the degradation of I*κ*B-*α* and reduced phosphorylated-I*κ*B-*α* level, compared to TNF-*α*-only-treated groups (*P* < 0.05, Figures 4 and 5). These results were consistent with our IF results. Moreover, the suppressive effects of 2-Cl-IB-MECA on TNF-*α*-induced NF-*κ*B activation occurred in a concentration-dependent manner (Figures 4 and 5). These results show that 2-Cl-IB-MECA inhibited TNF-*α*-induced NF-*κ*B activation.

### 3.5. Effects of 2-Cl-IB-MECA on TNF-*α*-Induced IL-8 and IL-1*β* Expression

**Figure 6:**
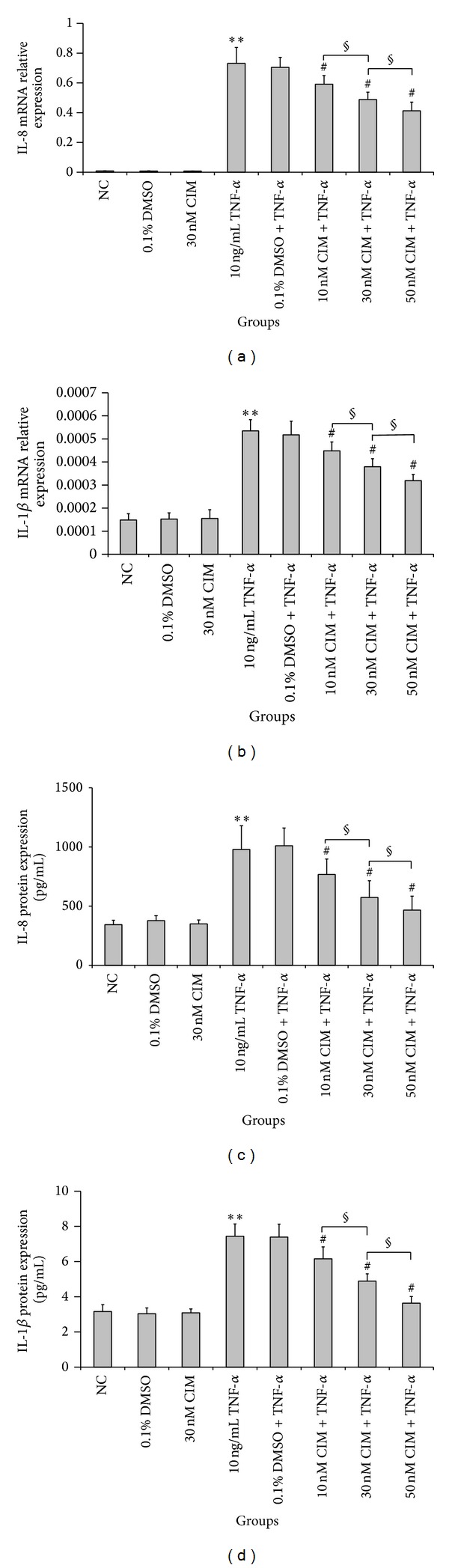
Effect of 2-Cl-IB-MECA on TNF-*α*-induced proinflammatory cytokine IL-8 and IL-1*β* expression in HT-29 cells. The cells were treated with various concentrations (10 nM, 30 nM, and 50 nM) of 2-Cl-IB-MECA for 30 min before TNF-*α* (10 ng/mL) stimulation and then incubated for 6 h in order to assay for mRNA expression by qRT-PCR or 24 h for measurement of protein secretion by ELISA. (a, b) The IL-8 and IL-1*β* mRNA were expressed at relatively low levels in the NC group. TNF-*α* treatment resulted in an increase in IL-8 and IL-1*β* mRNA expression. However, 2-Cl-IB-MECA significantly reduced the TNF-*α*-induced IL-8 and IL-1*β* mRNA expression in HT-29 cells in concentration-dependent manner. (c, d) In accordance with the alteration of mRNA, the TNF-*α*-treated group also had increased secretion of IL-8 and IL-1*β* proteins. Treatment with 2-Cl-IB-MECA significantly inhibited TNF-*α*-induced secretion of IL-8 and IL-1*β* protein in a concentration-dependent manner. Data are shown as the mean ± SD from three independent experiments. **P < 0.01 compared with the NC group; ^#^ *P* < 0.05 compared with the TNF-*α*-only-treated group; ^§^ *P* < 0.05 between indicated groups.

To investigate whether A3AR activation altered the production of proinflammatory cytokines IL-8 and IL-1*β* in HT-29 cells, we treated the cells with TNF-*α* (10 ng/mL) and various concentrations (10 nM, 30 nM, and 50 nM) of 2-Cl-IB-MECA. As shown in Figure 6, TNF-*α*-stimulation resulted in significantly increased IL-8 and IL-1*β* mRNA expression and secretion, compared to the NC group (*P* < 0.01). However, 2-Cl-IB-MECA significantly decreased TNF-*α*-stimulated IL-8 and IL-1*β* mRNA expression and secretion, compared to the TNF-*α*-only-treated group (*P* < 0.05). Moreover, the inhibitory effects of 2-Cl-IB-MECA on IL-8 and IL-1*β* expression occurred in a concentration-dependent manner (*P* < 0.05). These results demonstrate that 2-Cl-IB-MECA is an inhibitor of TNF-*α*-induced IL-8 and IL-1*β* expression in HT-29 cells.

Summary of the important results was shown in Table 2.

## 4. Discussion

A3AR is a subtype of the adenosine receptor family. Depending on the level of receptor activation, A3AR has been implicated in many pathophysiological processes [4]. A3AR appears to have a complex role, as both pro- and anti-inflammatory effects have been demonstrated. Forte et al. found that 2-Cl-IB-MECA can enhance TNF-*α* release from LPS-stimulated macrophages in an A3AR-dependent manner [17]. It has also been reported that A3^−/−^AR phenotype protected against DSS colitis in mice [18]. On the other hand, a protective role for A3AR has been reported. Human A3AR agonist thio-Cl-IB-MECA has an anti-inflammatory effect through the inhibition of proinflammatory cytokine expression including inducible nitric oxide synthase (iNOS), IL-1*β*, and TNF-*α* by modulating PI3K/Akt and NF-*κ*B signaling pathways in mouse macrophage RAW 264.7 cells and an* in vivo* mouse sepsis model [19]. Mabley et al. found that activation of the A3AR is effective in protecting against colitis, as IB-MECA protected DSS-colitis mice against colitis-induced inflammatory cell infiltration and damage and attenuated the increase in colon inflammatory cytokine and chemokine levels [20]. In a TNBS-induced rat colitis model, oral IB-MECA significantly prevented colitis-induced gene dysregulation, histopathology, gut injury, and weight loss, and IB-MECA or adenosine suppressed elevated free radicals in the* ex vivo* inflamed gut [21]. Besides, the anti-inflammatory actions of adenosine deaminase inhibitors against chronic established colitis depend on the sparing of endogenous adenosine, leading to enhanced adenosine A2A and A3 receptor activation [22]. Therefore, A3AR may be a new potential therapeutic target for colonic inflammation. Based on these findings, the role of A3AR in inflammation may depend on the systems investigated, the different experimental conditions used (e.g.,* in vivo* or* in vitro*), and the species examined [4].

It is well known that NF-*κ*B is an inducible transcription factor that mediates signal transduction between the cytoplasm and nucleus in many cell types including colonic epithelial cells [23]. NF-*κ*B family members control the transcriptional activity of various promoters of proinflammatory cytokines, cell surface receptors, transcription factors, and adhesion molecules that are involved in intestinal inflammation. The perpetual activation of NF-*κ*B in patients with active inflammatory bowel disease suggests that regulation of NF-*κ*B activity is a very attractive target for therapeutic intervention [24, 25]. Previous research has positioned NF-*κ*B as a central regulator of the intestinal epithelial cell innate immune response to infection with enteroinvasive bacteria by regulating the key components of the epithelial inflammatory gene program (i.e., IL-8, monocyte chemoattractant protein-1, TNF-*α*, cyclooxygenase-2, nitric oxide synthase-2, and ICAM-1) activated by the enteroinvasive bacteria [26].

In our study, we described for the first time the role and mechanism of A3AR in colonic epithelial cells by IF, qRT-PCR, and western blot. IF staining of cells for A3AR revealed that these receptors are mainly expressed on the cytomembrane, consistent with previous reports [2]. In addition, the expression of A3AR does not change due to TNF-*α* or 2-Cl-IB-MECA stimulation.* In vitro* study found that 2-Cl-IB-MECA was a potent A3AR agonist (Ki = 0.33 nM) with a 2500- and 1400-fold selectivity for A3AR versus A1AR and A2AR, respectively [27, 28]. The high selectivity of 2-Cl-IB-MECA enables us to study A3AR-mediated effects on inflammation, without the interference of the effects of other adenosine receptor subtypes. Therefore, we used 2-Cl-IB-MECA as an A3AR agonist in our study.

As evaluated by IF, qRT-PCR, and western blot analysis, TNF-*α* treatment resulted in phosphorylation and degradation of I*κ*B-*α* in HT-29 cells and the obvious nuclear translocation of NF-*κ*B p65. But activation of A3AR was able to reverse this phenomenon to some extent. We found that 2-Cl-IB-MECA counteracted the TNF-*α*-induced decrease in I*κ*B-*α* and inhibited phosphorylation of I*κ*B-*α*, leading to failed nuclear translocation of NF-*κ*B p65 in HT-29 cells in a concentration-dependent manner. Although it is reported that in the presence of IB-MECA (1 **μ**M), the percentage of apoptotic HT-29 cells significantly decreased [29] and Gessi et al. also found that 2-Cl-IB-MECA (10 **μ**M) was able to stimulate HT-29 cells proliferation through involvement of ERK1/2 kinases [30], in our study, examination of the cytotoxicity of 2-Cl-IB-MECA in HT-29 cells by using a CCK-8 assay indicated that, even at 400 nM, 2-Cl-IB-MECA did not affect the viability of HT-29 cells. Therefore, inhibition of TNF-*α*-induced NF-*κ*B activation by 2-Cl-IB-MECA with various concentrations (10 nM, 30 nM, and 50 nM), which were much lower than the previous publication, was not the result of a cytotoxic effect on these cells. Besides, the expression of p65, I*κ*B-*α* and phosphorylated-I*κ*B-*α* was not significantly different between the control group and the DMSO group indicating that the solvents used to dissolve 2-Cl-IB-MECA had no effect on NF-*κ*B activation. These results provided strong evidence that activation of A3AR plays a role in the suppression of TNF-*α*-induced NF-*κ*B activation. Furthermore, we found that the mRNA and secreted protein levels of IL-8 and IL-1*β* were increased in the TNF-*α*-treated groups but were inhibited in a concentration-dependent manner by 2-Cl-IB-MECA treatment. These data suggested that activation of A3AR mediated a reduction in the expression of downstream IL-8 and IL-1*β* in response to TNF-*α* stimulation in HT-29 cells. It is therefore conceivable that A3AR regulates TNF-*α*-induced inflammation by playing an anti-inflammatory role.

## 5. Conclusion

In summary, our findings indicated that A3AR is expressed and its activation has anti-inflammatory effect through the inhibition of NF-*κ*B signaling pathway associated with the inhibition of downstream IL-8 and IL-1*β* expression in human colonic epithelial cells. A3AR may therefore be a potential therapeutic target for the treatment of colonic inflammatory diseases such as inflammatory bowel disease.

## Figures and Tables

**Table 1:** Sequences of gene specific primers used for qRT-PCR.

Gene	Forward sequence (5′-3′)	Reverse sequence (5′-3′)
Human A3AR	GGCTGCCCTCAAATAACATC	CTCCACCTCTTCTTCACTTCTG
Human IL-8	GCAGAGGGTTGTGGAGAAGT	AACCCTACAACAGACCCACA
Human IL-1*β*	GGCAATGAGGATGACTTGTTCT	CTGTAGTGGTGGTCGGAGATTC
Human *β*-actin	CTGGCACCCAGCACAATG	CCGATCCACGGAGTACTTG

**Table 2:** Summary of the important results.

Items	Results
Cell viability	No significant effect on cell viability was observed at test concentrations of up to 400 nM (*P* > 0.05).
Location and qualitative expression of A3AR, NF-*κ*B p65, I*κ*B-*α*, and phosphorylated-I*κ*B-*α* in HT-29 cells treated with TNF-*α*, and/or 2-Cl-IB-MECA	TNF-*α*-stimulated cells pretreated with 2-Cl-IB-MECA showed no obvious change in A3AR expression. Pretreatment with 2-Cl-IB-MECA and subsequent stimulation with TNF-*α* attenuated NF-*κ*B p65 nuclear translocation and suppressed the phosphorylation of I*κ*B-*α*.
mRNA and protein expression of A3AR in HT-29 cells treated with TNF-*α* and/or 2-Cl-IB-MECA	The mRNA and protein expression of A3AR showed no significant change among the NC group, TNF-*α*-only treated group, and 2-Cl-IB-MECA + TNF-*α* groups (*P* > 0.05).
Effects of 2-Cl-IB-MECA on TNF-*α*-induced NF-*κ*B activation	Pretreatment with 2-Cl-IB-MECA prior to stimulation with TNF-*α* attenuated NF-*κ*B p65 nuclear translocation as p65 protein decreased in the nucleus of cells and increased in the cytoplasm, inhibited the degradation of I*κ*B-*α*, and reduced phosphorylated-I*κ*B-*α* level, compared to TNF-*α*-only-treated groups (*P* < 0.05)
Effects of 2-Cl-IB-MECA on TNF-*α*-induced IL-8 and IL-1*β* expression	2-Cl-IB-MECA significantly decreased TNF-*α*-stimulated IL-8 and IL-1*β* mRNA expression and secretion, compared to the TNF-*α*-only-treated group (*P* < 0.05)
